# Management of Cutaneous Squamous Cell Carcinoma of the Scalp in Kidney Transplant Recipients

**DOI:** 10.3390/cancers17071113

**Published:** 2025-03-26

**Authors:** Lucia Romano, Chiara Caponio, Fabio Vistoli, Ettore Lupi, Maria Concetta Fargnoli, Maria Esposito, Laura Lancione, Manuela Bellobono, Tarek Hassan, Elisabetta Iacobelli, Luca Semproni, Alessandra Panarese

**Affiliations:** 1 Department of General and Transplant Surgery, San Salvatore Hospital, ASL1 Abruzzo, Coppito, 67100 L’Aquila, Italy; lucia.romano@graduate.univaq.it (L.R.); fabio.vistoli@univaq.it (F.V.); laura.lancione@graduate.univaq.it (L.L.); mbellobono@asl1abruzzo.it (M.B.); 2 UOSD of General and Oncological Dermatology, San Salvatore Hospital, ASL1 Abruzzo, Coppito, 67100 L’Aquila, Italy; ccaponio@asl1abruzzo.it (C.C.); maria.esposito3@univaq.it (M.E.); 3 Department of Biotechnological and Applied Clinical Sciences, Via Giuseppe Petrini, University of L’Aquila, Coppito, 67100 L’Aquila, Italy; tarek.hassan@graduate.univaq.it (T.H.); elisabetta.iacobelli@graduate.univaq.it (E.I.); luca.semproni@student.univaq.it (L.S.); 4 Department of Maxillo-Facial Surgery, San Salvatore Hospital, ASL1 Abruzzo, Coppito, 67100 L’Aquila, Italy; ettore.lupi@graduate.univaq.it; 5 San Gallicano Dermatological Institute–IRCCS, Via Elio Chianesi 53, 00144 Rome, Italy; mariaconcetta.fargnoli@ifo.it

**Keywords:** cutaneous squamous cell carcinoma, kidney transplant recipients, skin tumors, scalp reconstruction

## Abstract

Background: Organ transplant recipients are at a significantly higher risk of developing skin cancer compared to the general population, particularly cutaneous squamous cell carcinoma. Approximately 3–8% of these carcinomas are located on the scalp. Scalp reconstruction is particularly challenging, especially for large excisions, due to the thickness of the scalp, the inelastic aponeurosis of the galea, and the integrity of the hair-bearing scalp. Additionally, in organ transplant recipients, the presence of numerous comorbidities and the increased risk of infection due to immunosuppressive therapy make management more complex. Based on our experience and the existing literature, we aim to describe possible reconstruction methods and discuss the combined management of medical and immunosuppressive therapy. Method: We present our experience with seven kidney transplant patients who underwent excision of cutaneous squamous cell carcinoma with a diameter larger than 3 cm. The crane technique involves three key steps. First, the tumor is excised with wide margins of disease-free tissue. Next, a pericranial flap is rotated and positioned to cover the exposed cranial bone. Finally, a bilayer dermal substitute is applied to create a microenvironment that supports skin graft implantation. Results: The crane technique was used for six patients. In one case, an O-Z rotation flap was used. All patients modified their immunosuppressive therapy, with those receiving antiproliferative therapy switching everolimus after surgery. Conclusions: When combined with a post-operative modification of the immunosuppressive regimen, the crane technique could be considered a feasible, safe, and effective approach to managing large cSCC of the scalp in fragile patients.

## Simple Summary

Organ transplant recipients are at significantly higher risk of developing skin cancer, particularly cutaneous squamous cell carcinoma, compared to the general population. These tumors are commonly found on the scalp. Scalp reconstruction, especially for large excisions, is complex due to the scalp’s thickness, the inelastic aponeurosis of the galea, and the need to preserve the integrity of the hair-bearing scalp. Additionally, organ transplant recipients face increased challenges due to comorbidities and the heightened risk of complications from immunosuppressive therapy. In this report, we present our experience with seven kidney transplant patients who underwent excision of cutaneous squamous cell carcinomas on the scalp with diameters greater than 3 cm. We highlight the advantages of the crane reconstruction technique and discuss the combined management of medical and immunosuppressive therapies. In cases of scalp cutaneous squamous cell carcinoma in transplant recipients, a multidisciplinary approach is essential for optimal outcomes.

## 1. Introduction

Renal transplantation provides the optimal long-term benefit for selected patients. Recent advancements in immunosuppression have reduced rates of allograft rejection and improved survival [1]. However, the duration of immunosuppression is associated with increased long-term complications [2]. Skin cancer is a serious complication that frequently affects solid organ transplant recipients. Immunosuppressive drugs are proposed to contribute to the risk through several mechanisms, including direct carcinogenic effects [3] and reduced immune surveillance [4]. Regardless of the agents used, the intensity and duration of immunosuppression appear to correlate with the risk of aggressive cutaneous carcinomas [5]. Advanced age, male sex, Caucasian ethnicity, pre-transplant malignancy, autosomal dominant polycystic kidney disease (ADPKD)-induced end-stage renal disease (ESRD), and immunosuppressive therapy with tacrolimus and mycophenolate mofetil (MMF) have been found to be independent risk factors for the development of skin cancers [6].

In the general population, skin cancer is a significant public health burden, with more than 1 million cases of nonmelanoma skin cancer (NMSC) in the United States each year [7]. These are also the most common types of skin cancer affecting kidney transplant recipients (KTRs), occurring at 65 to 250 times the frequency seen in the general population [8]. As many as 40% to 70% of transplant patients may eventually develop NMSC, with increased rates of cutaneous squamous cell carcinoma (cSCC) compared to basal cell carcinoma (BCC) [9]. These cancers follow a more aggressive clinical course in transplant recipients than in the non-immunosuppressed population. Some studies [10,11] have found that cSCC in solid organ transplant recipients is associated with greater tumor depth, a higher probability of recurrence, and a greater incidence of perineural or lymphatic invasion. Increased risk may also be associated with location on the ear, lips, and scalp [11].

The management of skin cancer in kidney transplant recipients (KTRs) requires a multidisciplinary approach [12], and surgical excision with post-operative margin assessment may be used in the treatment of higher-risk lesions [13]. Surgical excision and reconstruction may represent a challenge for surgeons, particularly in thick, inelastic anatomic zones such as the scalp. Its reconstruction is complex, especially in extensive and full-thickness defects involving the periosteum, bone, and dura mater [14]. Moreover, in organ transplant recipients, the numerous comorbidities, slowed wound healing, and increased risk of infection make the management of scalp lesions even more complex. Based on our experience and the literature, we aim to describe the possible reconstruction methods and the combined management of medical and immunosuppressive therapy.

## 2. Materials and Methods

At our transplant center, KTRs undergo regular dermatological and physical examinations every 3–6 months, allowing for early detection of skin tumors. Most cases of scalp cSCC are treated with excision and primary closure if the tumor is smaller than 3 cm in diameter.

Patients, included in the study, were consecutively enrolled over an 18-month period, from January 2023 to June For tumors larger than 3 cm, scalp closure required specific reconstruction techniques. Before surgery, all patients underwent a punch biopsy, confirming cSCC. They were then staged using an ultrasound of the head and neck lymph nodes, along with a chest CT scan.

In all cases, immunosuppressive therapy was adjusted approximately one month after surgery. Antiproliferative agents such as MMF were discontinued and replaced with an mTOR inhibitor, such as everolimus. Depending on disease staging, adjuvant therapy—including radiotherapy, chemotherapy, or immunotherapy—was considered.

All procedures performed in this study were in accordance with the ethical standards of the institutional research committee and with the 1964 Helsinki declaration. Each patient provided informed consent prior to the procedure.

### 2.1. Surgical Techniques

Two surgical techniques were used: The O-Z double rotation flap [15], with primary closure of the wound, was used in one case. In other cases, due to the extension of the resection (larger than 10 cm), a two-stage surgical procedure was planned (crane technique): the first step involved tumor excision with wide margins of disease-free tissue. In all cases, the specimen included a full-thickness resection. The periosteum was included in all cases. The scalp was elevated in the subgaleal plane circumferentially around the defect to allow full visualization of the pericranium. A wide pericranial flap, based on randomized blood supply, was harvested from the skull with a periosteal elevator. Next, the scalp was elevated with retractors, and back cuts were made in the pericranial flap with cautery or scissors to gain flap length as needed. The pericranial flap was rotated and fixated with absorbable sutures to cover the exposed cranial bone. If a single flap was insufficient for total coverage, a contralateral pericranial flap was elevated. The scalp was repositioned back to its original position without movement of the hairline. The surgical site was covered with a bilayer dermal substitute, which provides a microenvironment suitable for optimal tissue repair and wound preparation to support the implantation of skin grafts. A compressive dressing with a polyurethane sponge or medicated gauze was applied and secured with a bolster dressing. Patients were discharged the day after surgery and returned as outpatients after 4 days to remove the dressing. Dressing changes were repeated every 4–5 days. Twenty-one days after surgery, the dressing and the silicone layer were removed, and the integration of the deep layer was checked. In the second step, the definitive reconstruction was performed in all cases under local anesthesia, with a full-thickness skin autograft, after histological confirmation of oncological clearance [15,16].

### 2.2. Statistical Analysis

The characteristics of the study sample were analyzed using descriptive statistics. The study population was stratified into two groups, according to the absence or presence of recurrences. Discrete and nominal variables were expressed using frequencies and percentages, and Fisher’s exact test was used to examine differences between the groups. Statistical significance was set at *p* < 0.The data were processed using JASP Team (2024), JASP (Version 0.19.3) [Computer software].

## 3. Results

Approximately 70% of kidney transplant recipients (KTRs) undergo regular dermatological follow-up at our transplant center.

In our study, we included seven KTRs with scalp cSCCs larger than 3 cm in diameter who underwent surgical excision. The baseline characteristics of these patients are summarized in Table 1. The median age was 68 years (range 59–75), with six patients (85.7%) being male. The median time from kidney transplant to cSCC diagnosis was 11 years (range 2–20). All patients had scalp cSCC, with none presenting with lymph node or distant metastatic disease. Three patients had a pT1 tumor stage according to the TNM staging system (UICC/AJCC eighth edition, 2017) [17,18], one had a pT2, and three had a pT3 tumor stage.

All patients were on immunosuppressive therapy with a calcineurin inhibitor (CNI), specifically tacrolimus, combined with prednisone as part of their chronic immunosuppressive regimen (IS) prior to enrollment. Five patients (71.4%) were also receiving mycophenolate mofetil (MMF), while two (28.6%) were on therapy with everolimus. After surgery, all patients receiving MMF were switched to everolimus. The target blood levels of everolimus when used in combination with tacrolimus were between 3 and 5 ng/mL, with the combined target concentration of everolimus and tacrolimus being around 10 ng/mL.

Statistical analysis showed no significant differences between patients with and with-out recurrences in terms of anthropometric and preoperative characteristics (Table 2).

All patients underwent radical surgery, performed under local anesthesia combined with sedation. Given the increased risk of infection in immunosuppressed patients, antibiotic prophylaxis was administered with beta-lactam antibiotics for up to 7 days after surgery.

**Figure 1:**
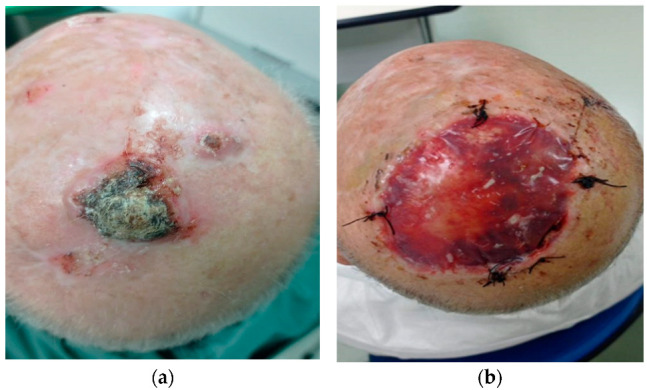

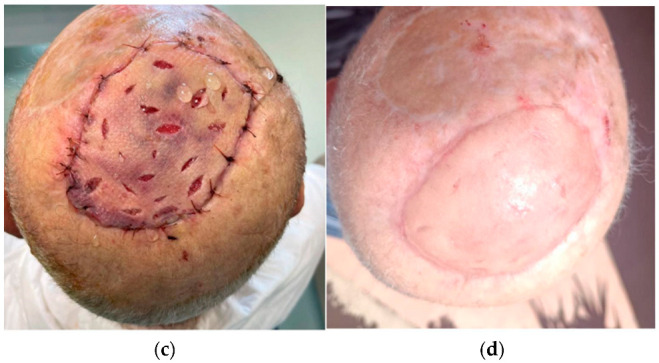
Crane technique. (**a**) SCC of the vertex; (**b**) pericranial flap with bilayer dermal substitute. (**c**) full-thickness skin autograft; (**d**) wound healing after one month.

Six patients underwent the crane technique, with final closure with a skin graft taken from the medial side of the right arm, which was performed one month later (Figure 1). In two of them, a new cSCC of the scalp developed immediately after the first operation, allowing for the rapid removal of a small lesion and closure by primary intention. In another one of these “crane technique” patients, histological examination revealed keratinizing cutaneous squamous cell carcinoma (cSCC), staged as pT3 according to the TNM system (UICC/AJCC eighth edition, 2017) [17,18] and T2 b according to Brigham and Women’s Hospital (BWH) criteria [19]. Due to multiple high-risk factors (BWH T2 b/T3), the patient received adjuvant radiation therapy at a total dose of 60 Gy, with 2 Gy daily fractions for 30 doses. Three months later, a new cSCC appeared at the periphery of the irradiated area. The patient underwent a second surgery to remove the neoformation, transposition of a galea flap, and placement of a dermal substitute. In this case, the galea flap experienced necrosis, possibly due to prior irradiation. After one month, a skin graft was performed from the medial side of the left arm, following the drilling of holes in the skull to expose the vascularization. The graft underwent necrosis in the central portion, while the peripheral section remained viable. Secondary intention healing was achieved over three months.

**Figure 2:**
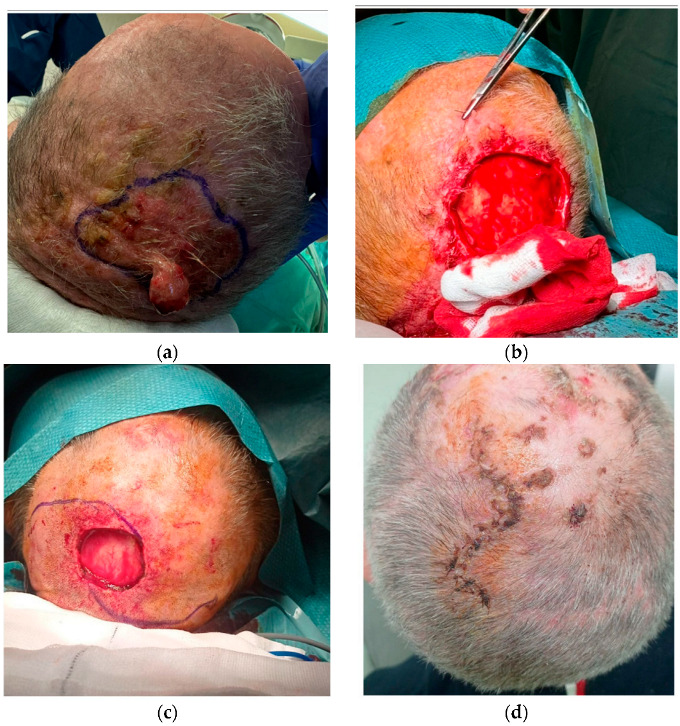
O-Z flap technique. (**a**) SCC of vertex; (**b**) exeresis of SCC.; (**c**) O-Z flap set up for reconstruction of defect; (**d**) wound after 10 days.

One patient was reconstructed using an O-Z double rotation flap, achieving primary closure of the wound. An extemporaneous histological examination was necessary during surgery to confirm the radicality of the excision before proceeding with closure (Figure 2).

All patients received modifications to their immunosuppressive therapy. Mycophenolate mofetil (MMF) was replaced with everolimus, with a target trough level of 4–6 ng/mL, and patients continued prednisone (5 mg) and tacrolimus, with target trough levels of 4–6 ng/mL. The patients were followed with dermatological clinical and physical examinations every 3–6 months and with a neck ultrasound every 6 months. After a mean follow-up period of 8 months (range 6–24 months), a total of two new cases of cSCC were observed.

## 4. Discussion

In a recent real-world retrospective analysis, non-melanoma skin cancers (NMSCs) were identified as the most common cancers among post-kidney transplant patients [19]. Risk factors for the development of NMSCs include ultraviolet radiation, age, ethnicity, hereditary syndromes, infections (particularly certain subtypes of papillomavirus), and a family history of cutaneous cancer [20]. Immunosuppressed patients face an increased risk of developing more aggressive forms of cutaneous squamous cell carcinoma (cSCC) [21], suggesting that immunosurveillance plays a critical role in the progression and control of these cancers [22]. Specifically, cSCC in solid organ transplant recipients tends to be associated with greater tumor depth, higher recurrence rates, and increased incidence of perineural or lymphatic invasion [9,10,11].

Given the elevated risk of NMSCs, their potential for atypical presentations, and the poorer outcomes in immunocompromised individuals, clinicians should maintain a high level of suspicion, particularly for cSCC. All cSCC in these patients should be treated as ’high-risk’ in terms of management strategies [20]. Complete surgical resection is the primary treatment for operable tumors, and Mohs surgery, or complete circumferential peripheral and deep margin assessment, are often recommended [23]. Radiotherapy remains an important option when surgery is not feasible [20]. Current evidence suggests that immunotherapy with immune checkpoint inhibitors (ICIs) should be considered the preferred treatment for transplant recipients with advanced cSCC when surgery, radiotherapy, or both fail, and it should be considered a first-line therapy before chemotherapy [24,25]. However, the use of PD1-blockade increases the risk of allograft rejection, with approximately 40% of grafts being rejected, based on data from 40 organ transplant recipients with metastatic cSCC [20].

A multidisciplinary approach is essential for managing the most challenging cases. Surgical excision and reconstruction may pose difficulties, especially in thick, inelastic anatomic zones like the scalp [14], where approximately 3–8% of cSCC are typically located [26]. Several surgical techniques are available for scalp reconstruction, including primary closure, local or regional flaps, free tissue transfer, and tissue expansion [27]. Key factors in selecting the appropriate technique include defect thickness, size, location, pericranial status, prior surgical procedures, and the patient’s overall medical and functional status [28]. In organ transplant recipients, numerous comorbidities, delayed wound healing, and an increased risk of infections further complicate the management of scalp lesions.

From our results, anthropometric and clinical characteristics were comparable between groups with and without recurrences, suggesting that there were no preoperative confounding factors regarding the risk of recurrence. All cases of recurrence were T3 tumors, but this data did not reach statistical significance.

In this unbiased sample, we described the advantages of the crane reconstruction technique and the combined management of medical and immunosuppressive therapy. The original goal of the crane principle, proposed by Millard in 1969, was to shorten the interval of pedicle division of the abdominal flap in hand reconstruction [29]. Ship et al. and Wolfe later extended the crane principle from hand surgery to scalp reconstruction [30,31].

In our cases, reconstruction was carried out in six cases with transposition of a galea flap, placement of a dermal substitute, and closure with a skin graft, and in one case with an O-Z rotation flap. Our purpose was to support the advantages of the crane technique, in line with what has been reported in the literature, and to propose it as the technique of choice in cases of large scalp SCC in fragile patients. This approach offers the key advantage of being performed under local anesthesia, which is particularly important for patients with compromised general conditions, for whom general anesthesia is often contraindicated. Additionally, the reconstruction is surgically rapid and minimally invasive. The excision margins are left in place and can be easily reworked if recurrence or involvement occurs. Given the general tendency of transplanted patients to experience skin cancer relapses, performing an extemporaneous histological examination during surgery can be considered a standard practice to ensure the radicality of the removal. However, this may significantly extend the operating time. Finally, the crane technique allows for the positioning of the dermal substitute on well-vascularized tissue, rather than on an area that may be compromised by the tumor itself or by prior radiation therapy.

Some potential disadvantages of this technique include suboptimal cosmetic outcomes and the need for the patient to undergo a two-step procedure.

In addition to treating the neoplasm, modifying the immunosuppressive regimen after a cancer diagnosis aims to restore the antitumor immune response, thereby improving the oncological prognosis.

A multidisciplinary consensus conference on the management of kidney transplant patients with cancer expressed uncertainty regarding whether switching from CNI to everolimus improves patient or graft survival in KTRs with metastatic non-cutaneous cancer who are not undergoing chemotherapy [32].

From the literature review, it emerged that the CONVERT trial [33] demonstrated a lower incidence of malignancies at 12 and 24 months in kidney transplant recipients (KTRs) who were converted to sirolimus, compared to those maintained on calcineurin inhibitors (CNIs). Another systematic review [34] showed that the use of sirolimus was associated with a 40% reduction in the risk of malignancy and a 56% reduction in the risk of developing nonmelanoma skin cancer (NMSC) compared with controls. Additionally, a systematic review of 24 studies reported a 49% reduction in cancer incidence in patients treated with mTOR inhibitors (mTORi) [35].

However, it has been reported that the use of mTORis in KTRs undergoing oncological surgery can slow wound healing and is associated with other surgical complications, with an incidence ranging from 15% to 32% after kidney transplantation [36]. For this reason, it is recommended to modify or suspend mTORis before surgery to help prevent perioperative complications, and to reintroduce it after wound healing.

Therefore, the management of KTRs always requires a multidisciplinary approach, customized based on individual risk factors such as rejection, renal function, life expectancy, and comorbidities. Our study has a small sample size as its main limitation; however, the described cases are rare, as they involve kidney transplant recipients with a vertex cSCC greater than 3 cm. All other cases of cSCC were excluded from this study. Nevertheless, our results are consistent with those reported in the literature.

## 5. Conclusions

In cases of cSCC of the scalp, several surgical techniques are available, but many factors have to be considered. In organ transplant recipients, numerous comorbidities, slowed wound healing, and an increased risk of infection make managing scalp lesions even more complex. The “crane technique” offers several benefits: it can be performed under local anesthesia, is rapid and non-invasive, and leaves the excision margins in place, which allows for easy reworking in case of recurrence or involvement. Furthermore, it positions the dermal substitute on well-vascularized tissue that has not been damaged by the tumor itself or by radiation therapy. Therefore, when combined with a post-operative modification of the immunosuppressive regimen, the crane technique could be considered a feasible, safe, and effective approach to managing large cSCC of the scalp in fragile patients. Among our future goals is to enlarge the study sample.

## Figures and Tables

**Table 1:** Patients’ characteristics.

Variable	Patients (N = 7)
Age, years, median (range)	67.86 (59–75)
Male sex, n (%)	6 (85.7)
Caucasian ethnicity	7 (100)
Original Kidney disease, n (%)	
Diabetes mellitus type II	1 (14.3)
Glomerulonephritis	2 (28.6)
ADPKD	1 (14.3)
Unknown disease	3 (42.9)
Pre-transplant malignancies, n (%)	
Previous cSCC	1 (14.3)
None	6 (85.7)
Comorbidities, n (%)	
Diabetes mellitus type II	1 (14.3)
Arterial hypertension	7 (100)
Duration of dialysis treatment prior to transplantation, months, mean (range)	68 (17–96)
Time since kidney transplant, years, median (range)	11 (2–20)
Immunosuppressive (IS) regimen before enrollment, n (%)	
Tacrolimus	7 (100)
Everolimus	2 (28.6)
MMF	5 (71.4)
Prednisone	7 (100)
Induction immunosuppression, n (%)	
Methylprednisolone 500 mg and Basiliximab 20 mg e.v.	7 (100)
Immunosuppressive (IS) regimen after surgery, n (%)	
Tacrolimus	7 (100)
Everolimus	7 (100)
MMF	0 (0)
Prednisone	7 (100)
Histological type, n (%)	
Cutaneous Squamous cell carcinoma	7 (100)
Tumor stage ^1^, n (%)	
pT1	3 (42.9)
pT2	1 (14.3)
pT3	3 (42.9)
pT4	0
Nodal stage ^2^, n (%)	
N0	7 (100%)
Metastatic disease ^2^, n (%)	
M0	7 (100%)
BWH tumor classification, n (%)	
T1 0 Hight-risk factors ^4^	1 (14.3%)
T2 a 1 Hight-risk factors	5 (71.4%)
T2 b 2−3 Hight-risk factors	1 (14.3%)
T3 4 Hight-risk factors or bone invasion	0
Stage ^3^, n (%)	
Stage I	3 (42.86%)
Stage II	1 (14.3%)
Stage III	3 (42.86%)
Stage IVA-IVB	0
Relapse or new scalp tumours, n (%)	3 (42.86%)
Adjuvant radiation, n (%)	1 (14.3%)

ADPKD: autosomal dominant polycystic kidney disease; cSCC: cutaneous squamous cell carcinoma; MMF: mycophenolate mofetil; BWH: Brigham and Women’s Hospital tumor classification. ^1^ UICC/AJCC staging 8th edition, 2017. ^2^ AJCC staging 8th edition. ^3^ BWH, Brigham and women’s hospital; ^4^ The identified high-risk factors in BWH classification include tumor diameter of 2 cm or greater, poorly differentiated histological type, perineural invasion of nerves measuring 0.1 mm or more in diameter, or tumor invasion extending beyond subcutaneous fat (excluding bone invasion, which elevates the tumor to BWH stage T3).

**Table 2:** Correlation between recurrence of cSCC and patients’characteristics.

	Recurrence (n = 2)	No Recurrence (n = 5)	*p*-Value
Age ≥ 67 years-old			1.000 *
Yes	1 (50)	3 (60)	
No	1 (50)	2 (40)	
Male sex, n (%)	2 (100)	4 (80)	1.000 *
Time since kidney transplant ≥ 12 years			1.000 *
Yes	1 (50)	3 (60)	
No	1 (50)	2 (40)	
Tumor stage, n (%)			0.600 *
pT1	0	2 (50)	
pT2	0	1 (25)	
pT3	2 (100)	1 (25)	
Immunosuppressive regimen witheverolimus before enrollment	1/1	1/4	1.000 *

* Fisher’s exact test.

## Data Availability

Data are unavailable due to privacy. Data are available upon request.

## Abbreviations

The following abbreviations are used in this manuscript:

BCC	Basal Cell Carcinoma
BWH	Brigham and women’s hospital
CNI	Calcineurin inhibitor
CTS	Computer Tomography Scan
cSCC	cutaneous Squamous Cell Carcinoma
ICIs	Immune Checkpoint Inhibitors
IS	Immunosuppressive
KTRs	Kidney Transplant Recipients
MMF	Mycophenolate Mofetil
mTORi	mammalian target of rapamycin inhibitor
NMSC	Non Melanoma skin cancer
